# Commodification of care and its effects on maternal health in the Noun division (West Region – Cameroon)

**DOI:** 10.1186/s12910-018-0286-1

**Published:** 2018-06-15

**Authors:** Ibrahim Bienvenu Mouliom Moungbakou

**Affiliations:** Department of Sociology and Anthropology, University of Maroua, Maroua, Cameroon

**Keywords:** Commodification of care, Maternal health, Noun division, Maternal health services

## Abstract

**Background:**

Since the mid-1980s, there has been a gradual ethical drift in the provision of maternal care in African health facilities in general, and in Cameroon in particular, despite government efforts. In fact, in Cameroon, an increasing number of caregivers are reportedly not providing compassionate care in maternity services. Consequently, many women, particularly the financially vulnerable, experience numerous difficulties in accessing these health services. In this article, we highlight the unequal access to care in public maternity services in Cameroon in general and the Noun Division in particular.

**Methods:**

For this study, in addition to documentary review, two qualitative data collection techniques were used: direct observation and individual interviews. Following the field work, the observation data were categorized and analyzed to assess their relevance and significance in relation to the topics listed in the observation checklist. Interviews were recorded using a dictaphone; they were subsequently transcribed and the data categorized and coded. After this stage, an analysis grid was constructed for content analysis of the transcripts, to study the frequency of topics addressed during the interviews, as well as divergences and convergences among the respondents.

**Results:**

The results of this data analysis showed that money has become the driving force in service provision. As such, it is the patient’s economic capital that counts. Considered “clients”, pregnant women without sufficient financial resources wait long hours in corridors; some die in pain under the indifferent gaze of the professionals who are supposed to take care of them. In sharp contrast, the findings revealed that financially privileged patients are able to bribe caregivers to attract their favour and obtain prompt, careful, and effective care.

**Conclusion:**

These ethical abuses observed in the Noun public health facilities drive women to use, from the beginning of their pregnancies to the delivery, only healthcare delivered by traditional health attendants.

## Background

After the fall of world prices of its main cash crops (coffee, cocoa, etc.) in the mid-1980s, Cameroon entered into an unprecedented economic crisis. Resources to finance major projects became scarce, and people, mostly farmers, fell back into extreme material poverty. This new environment characterized by cash problems compelled the country to acquiesce to the joint request of the International Monetary Fund (IMF) and the World Bank (WB)—the structural adjustment programmes (SAPs)—to revamp its economy [1]. On the health front, the government’s inability to continue providing free care obliged it to ratify the Bamako Initiative to reduce all forms of temporary or permanent exclusion in health services [2].

Both policies, whose stated objectives had been the return to economic growth and the strengthening of the health system, respectively, ultimately encouraged the generalization of certain deviations^1^ in public governance in Cameroon. In administrative services, for instance, the reduction of civil servants’ salaries in 1994 led to the exacerbation of ethical problems such as absenteeism and demotivation at work, favouritism, clientelism, neopatrimonialization of public goods, and commodification of services [3]. As the government became powerless to contain the expansion of unethical anti-values in the institutions responsible for implementing public policy, those institutions were transformed into a vast market where service providers and service requesters are engaged in several types of transactions [4]. For example, as described by Ondoua [5], in those services financial transactions have become the main means of obtaining even the slightest service.

Health services have not been spared by this new way of ‘serving’ that quickly placed Cameroon among the most poorly governed countries in the world [4]. In these services which—as required by international treaties ratified by Cameroon—are supposed to provide care regardless of gender, ethnicity, race, religion, political views, or social class, ethical problems were in fact exacerbated. Thus, the redirection of patients from formal to informal care centres, the diversion and resale of drugs—officially free to pregnant women—and especially the trading of health care services, all became widespread practices in health facilities [5–8].

These negative practices, which have transformed Cameroonian public hospitals into a vast market, have been normalized to the extent that care seekers are seen as clients routinely expected to enable service providers, through payment of extra fees, to make ends meet. As a result, although public hospitals continue to attract many patients because of their theoretically ‘affordable’ official costs, access to healthcare—even the most essential—now depends on the patient’s financial resources [3]. As such, patients who are financially vulnerable are not welcomed. An illustrative example of this transformation of healthcare into a commodity is the case of these mothers who, as Djoko reported, are aligned each morning in front of Africans hospitals in the total indifference of the caregivers, supporting the cries of their children [4]. These health professionals’ behaviours, although contrary to medical deontology, are so embedded in Cameroonian public hospitals that patients who are unable to pay extra fees for care die every day.

Such trade in healthcare, among other deviant practices that have caused Cameroon to be ranked twice (1998, 1999) by Transparency International as the most corrupt country in the world, has been addressed by several authors [4, 7, 9]. However, careful review of this literature shows this problem has long been mainly investigated by journalists, political scientists, and economists. Olivier de Sardan et al. confirmed this by emphasizing that, until the early 2000s, there had been very few socio-anthropological studies examining how the functioning of public health facilities has slipped away from official codes and standards [10].

To enrich the existing literature, this paper exposes the problem of the commodification of healthcare and the medical ethics violations that occur in public health institutions and specifically their impact on maternal health in the Noun Division, a locality with the highest composite fertility index in the West Region of Cameroon [11]. Two questions guided our research: 1) What strategies have service providers developed to generate extra revenue in healthcare facilities of the Noun Division? 2) How do service providers’ non-compliant attitudes influence antenatal care and facility-based deliveries in the Noun Division?

## Methods

### Data collection

The results presented in this article are based on a qualitative study carried out between May and July 2016 in the Foumban, Foumbot, Bangourain, and Kouoptamo health districts of the Noun Division. These were chosen for their sizeable populations and, more specifically, their high rates of maternal mortality. In addition to documentary review, data were collected using a combination of two qualitative techniques: direct observation and interviews. Authorization for the study was issued by the Ethics Committee of the West Regional Public Health Delegation. Given the sensitive and delicate nature of the care delivery problem [7, 12], we used the direct observation method, which consisted in mingling with patients [10] and pretending to be attendants of expectant women. Such immersion in the health services of the target localities enabled us to observe passively, using a protocol elaborated prior to the field work, how financially vulnerable pregnant women and mothers were received, how long they stayed in the waiting rooms, and how they were treated by maternal healthcare providers.

Individual interviews were conducted with 11 service providers (men and women), 13 pregnant women, and five mothers of 0-to-5-year-old children, all selected through a purposive approach, which consists in selecting research participants whose profile corresponds to a set of predefined characteristics. These three groups of actors constituted the key protagonists in the issue being investigated; as such, their participation, opinions, attitudes, and comments were considered to be of prime importance to the data collection and analysis. To contact service providers, we obtained from the managers of the four health districts the list of midwives and gynaecologists working in their localities. After we had presented, by phone, our research project to 20 of them, seven midwives and four gynaecologists (two males and two females) accepted to be interviewed. Others declined, because of the sensitive nature of the subject. Arranging interviews with pregnant women and mothers was facilitated by community health workers (CHWs). Thanks to their proximity to the populations, the CHWs were able to contact more than 30 women willing to be respondents in the research. Some were unavailable because of their schedules, but ultimately we were able to interview 19 women (13 pregnant women and five mothers of 0-to-5-year-old children) coming from both privileged and vulnerable socioeconomic backgrounds. The topics discussed with all these respondents included: the reception given to maternity services users; the provision of antenatal care; the management of obstetric emergencies and drugs; relationships between service providers and care seekers; and strategies used by service providers to generate additional revenue.

### Data analysis

The data were analyzed differently in accordance with the data collection techniques: direct observation and interviews. Data collected through direct observation were noted in a field journal. After the field work, the data were categorized and then analyzed based on the relevance of their content and their significance in relation to the topics on the observation checklist. With regard to data collection from interviews, the sessions were recorded using a dictaphone, and those recordings were subsequently transcribed. The various points were then categorized and codes were assigned to those that were similar. After coding, an analysis grid was constructed to serve as a basis for analysis of the corpus. In the content analysis of the transcripts, the frequency of topics addressed during the interviews was studied, as well as divergences and convergences among different participants’ reports. The data analysis was guided by the above-mentioned themes: the reception given to maternity services users; the provision of antenatal care; management of obstetric emergencies and drugs; relationships between service providers and care seekers; and strategies used by service providers to generate additional revenue.

## Results

### Commodification of healthcare facilities in the noun division: Service providers’ strategies

Commodification, or illegal trade that places financial resources at the heart of transactions between service providers and care seekers, is widespread in public hospitals of the Noun Division. More often practised by providers exercising their ‘power’ to provide care, this type of transaction, condemned by article 13 of the Prime Ministerial Decree No. 2004/321 of 08/12/2004 on the Codes of ethics of care givers,^2^ is common in public maternity units, taking the form of diversions of users, drugs, and some materials, as well as extortion of patients, etc. Diversion of users, for instance, consists in redirecting pregnant women intercepted in public health facilities to private or informal care centres. This is mostly practised by providers who occupy positions of power. These are health district managers, hospital directors, medical specialists, and integrated health centre (CSI) managers, who transform their homes into informal healthcare centres. They spend the greater part of their work time in these centres, regularly using material diverted from public institutions. That is what a midwife inferred: “*It is difficult to find the hospital director here because, as a gynaecologist, he consults most of the time at home. I suppose those consultations give him extra money*” (Respondant#1 (R1)). To justify that professional behaviour, which contravenes what is officially prescribed in the Decree cited above, a gynaecologist says: “*As you might know, medical doctors are not well paid in our country. We work every day and night to help pregnant women, but the income we receive is not proportionate. So we’re obliged to consult in private. It’s the only way to supplement our income*”. (R2).

In other cases, instead of redirecting pregnant women to their homes, these maternal health professionals refer them, for better monitoring, to private health facilities where they work part-time. During this period of support, which extends from the third month of pregnancy to the delivery, they do prenatal consultations with pregnant women from privileged classes, perform ultrasounds, and assist them when they have health problems. For childbirth, those who practise in private care centres that have a fairly well-developed technical platform do not hesitate to refer their clients there. A provider in the Foumbot neighbourhood confirmed this practice: “*When women I follow during their pregnancies are ready to deliver, I refer them to my friend’s clinic, where I work when I’m not here. They have appropriate materials to facilitate delivery*”. (R3).

However, when the private facility is not sufficiently equipped to ensure safe delivery, they bring their clients to the public maternity hospitals, where they mobilize human resources or colleagues to ensure a safe delivery. In this environment, usually marked by racketeering, extortion, and theft [7], any health workers on duty who would refuse to care for these referred women would find themselves in a professional conflict with those women’s protectors. Speaking about this practice, which significantly cuts into the working time of maternal health workers and dilutes the quality of care administered in public maternity units, a mother who had just been delivered said:

*When I went for consultation [at the hospital] during my last pregnancy, the doctor who received me took good care of me. Then, when I was leaving, she said, ‘Madam, the time you spent waiting outside is not good for your condition. If you wish, you can come to my clinic for your next prenatal visits. That way, I’ll take good care of you.’ For me, it was an opportunity I had to seize in order to be well attended. So, until the ninth month, I made my visits without pressure at this doctor’s clinic. I paid more, but it was much better than putting up with the indifference of some nurses at the public hospital. When contractions started, my husband called her, and she came quickly and took me to the public hospital. There, she mobilized her colleagues to make sure I gave birth under better conditions. We kept in touch. For future deliveries, I would go directly to her.* (R4).

While these women are well-attended because of their financial privilege, pregnant women of the common classes are abandoned to the control of frustrated public sector employees who cannot do anything else to generate additional incomes. During our survey, in a visit to a health centre owned by a service provider in Mambain, a small populous neighbourhood in Foumban, we were able to observe this ethical drift that produced unequal access to maternal healthcare. Upon arrival at the mini-centre in this practitioner’s home, we counted 30 pregnant women, who were mainly, according to him, wives of civil servants, traditional authorities, and businessmen. To justify such crowds in his place, the owner assured us that the pregnant women were better monitored and that the risk of error in their exams was near zero.

This personalization of maternal health—which has given rise to proprietary expressions such as my ‘pregnant woman’, ‘my patient’, and even ‘my son’s mother’—is even more pronounced in rural areas. Some CSI managers spend their whole day consulting at home. Others desert their duty post for several days to work in private care offices in town. In Bangourain, we found two CSIs closed, with a notice on the door: ‘The practitioner is absent and will return next week’. In another locality, we observed that the practitioner received more than half of pregnant women at home. A gynaecologist explained this shift of care provision away from official standards: “*At the hospital, there are so many patients that I cannot take care of all them. Then those who need special monitoring take a bed here. It allows me to make a little more money because the government doesn’t give us very much*” (R5). Patients who are unable to take a bed in this practitioner’s home remain at the CSI, waiting and hoping he might visit. During these waiting times, which are generally very long, some go back and others die in a state of fatigue and pain in the corridors.

The diversion and resale of drugs that are officially free, a very common phenomenon in the Noun Division, is practised by providers across the chain of care. We observed that in both urban and rural health facilities, midwives, nurses, and community pharmacists each had a private pharmacy containing drugs and other materials diverted from the official outlets or even from patients’ hospital rooms. They offered them directly to patients in consultation rooms at bargain prices. Some are even further motivated by their greed to create artificial shortages in pharmacies, thereby forcing users to turn to them or to their partners’^3^ place. A care provider in Bangourain attempted to justify this mismanagement of drugs, on the pretext that “*the salaries of care providers are very low. Those who don’t have the opportunity to consult elsewhere are forced to divert drugs and sell them in the informal sector to have a decent life*”. (R6).

During a quasi-observation in a public care centre in Bangourain on July 13, 2016, we were able to observe the *modus operandi* of this practice, which contravenes medical ethics. During working hours, service providers take advantage of patients’ weakness and inattention to the care being administered to them to extract drugs from their pharmaceutical shelf. When patients have come accompanied by attendants, those persons are simply asked to wait in the corridor, and in their absence the service providers carry out their plan. As this new mother related:

*After my delivery, I lost a lot of blood. Because I was worried, the doctors decided to keep me for a few days. But each time I was made to buy some drugs, they always disappeared. In the end, I realized that the drugs were stolen from me when my sister, who assisted me, was sent out during patient rounds. It’s a disgrace.* (R7).

Beyond these two practices that undermine the hospital’s income, the maternity users are daily victims of racketeering in the Noun Division. As seen in several childbirth services at the national level, they are regularly forced to pay fees that have no official reference. To meet a gynaecologist for pre- and postnatal visits, for example, the parturient or young mother is obliged to pay fees beyond the official fees that go to the hospital’s account. Amounting to 2 USD, this added payment that opens the gynaecologist’s door goes straight into his pocket. Sometimes the persons mediating these exchanges also demand a supplement that goes into their own accounts, as described by this pregnant woman:

*When you arrive at the hospital for prenatal visits, you’re obliged to pay additional charges to meet the doctor who is supposed to see you—otherwise you won’t be seen. And because in our district hospital there’s only one doctor to consult with pregnant women, we are sometimes obliged to bribe the secretary so that she introduces you to her. Since it is important to know how the baby is in the womb, we have no choice.* (R8).

Twelve women out of 19 interviews confirmed they had paid additional amounts to meet the doctor during their pre- and postnatal visits. While health workers cannot demand illegal payments from these women, they expect bribes. This practice, different from racketeering, offers the pledge of prompt care and closely monitoring in the Noun Division’s health facilities. In public maternity units, for example, pregnant women, regardless of their morbid condition, do not easily engage care providers’ sensitivities when the latter are not financially motivated. To be examined or brought to the delivery room on arrival, it is imperative that these women and their relatives give *cola* or *pay taxi* to practitioners. If not, they languish in the waiting room while those who are supposed to save them discuss private affairs, watch movies, or phone relatives.

This indifference towards vulnerable parturients was observed on July 19, 2016, in a public maternity unit in Kouoptamo neighbourhood. When a car with a woman on board arrived at the health facility, her husband and other relatives rushed to support her. They took her to the maternity services, but the providers on duty pretended it was the end of their working day and refused to admit the woman. While the husband was arguing with them, a woman who had just given birth whispered to another family member: “*Give them something and they’ll take care of her quickly*.” Whereupon the pregnant woman’s husband went to one of them and discreetly handed over the sum of 10 USD. A few minutes later, a midwife came back smiling, took the parturient and brought her into the room, where she delivered less than 5 minutes later. The new mother^4^ later said, “*In this hospital, you can’t receive the slightest care if you haven’t motivated [bribed] the caregivers*”. (R9) Another added, “*You can die if you don’t have enough money when you get to the hospital because, besides the official fees, you have to bribe doctors so they’ll take good care of you*”. (R10).

### Commodification of maternal healthcare and its effects in the noun division

The commodification of maternal healthcare has serious health consequences for the women of the Noun Division. Moreover, in the opinion of one service provider interviewed at the Regional Health Delegation, this division is the one with the highest number of women dying in childbirth. The subsections below present the impacts on prenatal consultations and institutional deliveries of this blatant violation of medical ethics.

### Commodification of maternal healthcare and its effects on prenatal care in the noun division

Antenatal visits—conducted by gynaecologists, midwives, or general practitioners—are the healthcare services administered to women throughout their pregnancy until delivery. Their purpose is to monitor the course of pregnancy and detect any abnormalities that may occur during its evolution. The World Health Organization (WHO) recommends that all pregnant women should undergo at least four prenatal visits. In the Noun Division, access to these visits, whose importance for maternal and fetal health is well established, remains a prerogative of women who have the economic capital to facilitate access to health institutions. This restriction to privileged women has been verified in our work. For example, one practitioner working in Foumban central hospital reported:

*Here, very few women do antenatal visits, despite the intensive communication we carry out in neighbourhoods, in ceremonies and places of prayer, in the markets and even over community radio. A few start but don’t do all four visits, as recommended by the national protocol.… I think the lack of financial resources has something to do with it. Here, women are mostly dependent on their husbands, who are poor. It’s difficult to get them [husbands] to understand that antenatal care is important for both the mother’s and the unborn baby’s health. So they consider antenatal care as useless spending.* (R11).

Also, the main recipients—women of reproductive age—think antenatal care providers’ indifference towards vulnerable women and racketeering of care seekers are rampant, despite the public display of messages against corruption on all the hospital notice boards. This is why many of them prefer going to the delivery without having sought any antenatal care. In case of any health problem, they prefer going to CHWs, who provide care without the threat of violence and at low prices. Others, like this respondent, prefer to rely on God:

*For prenatal visits here, we must have not only time but also money. When we get to the hospital, not only do the midwives keep us waiting a long time, but when the care begins each person involved wants us to give him something. When I went there last month for my second visit, I spent about 4000F (about 8 USD). I don’t know if I’ll go next trimester because my husband and I have nothing. We barely manage to get by. I really hope God will allow me to give birth in good conditions.* (R12).

These observations show that the requirement for additional payments is the main reason for the low consumption of antenatal care services in the Noun Division. The vast majority of women interviewed in this study confirmed this, pointing an accusing finger at a system in which multiple payments are extorted along the pregnant women’s hospital trajectory. The above-cited conclusion of R12 during her interview—“*I don’t know if I’ll go next trimester because my husband and I have nothing. We barely manage to survive*”—gives a more detailed account. These vulnerable women who do not use healthcare facilities during their pregnancies settle for recipes concocted in the community, based on indigenous knowledge, by elderly women renowned for their maternal healthcare experience. A woman who had just given birth affirmed the efficiency of these traditional healthcare providers:

*I didn’t go to the hospital even once when I was pregnant. Whenever I had a health problem, I ran quickly to see a mother who lives in the neighbourhood. And I gave birth without any problems. When I arrived at the hospital the day of delivery, the nurses were surprised and disappointed to know that I hadn’t had even one prenatal visit.* (R13).

If the commodification of maternal healthcare restricts the rate of antenatal care use in public health facilities of the Noun Division, this prompts a new question: how does this ethical drift that introduced discrimination in public health institutions affect facility-based deliveries in this part of Cameroon?

### Effects of commodification of maternal healthcare on facility-based deliveries

Not only does the commodification of maternal healthcare impede access to prenatal care in the Noun Division, it also inhibits facility-based deliveries. Despite efforts made by officials in this area to improve the geographical coverage of maternal healthcare and to communicate about the dangers of home delivery, many women continue to use the services of traditional birth attendants. This means very few women give birth with the assistance of a health professional. This has been confirmed by statistics coming from the West Regional Public Health Delegation. In an interview, one staff member said, *“With a facility-based birth rate of 54%, Noun is the Western division where women are least assisted by a health professional during childbirth”* (R14).

This statement was confirmed by a maternal healthcare provider working in a Kouoptamo hospital:

*In this hospital, we certainly record deliveries regularly, but many women still give birth in the community. I can even say that those who deliver at home are more numerous than those who come to the hospital, despite sensitization activities.* (R15).

Our respondents offered a variety of reasons for the high prevalence of unassisted deliveries recorded by officials. However, in the content analysis of interviews, the supplementary payments required for obstetrical services emerged as the most important explanatory factor. Indeed, many respondents pointed to the high cost of care in public hospitals and the preferential care provided to financially privileged persons. That is exactly what one respondent in Foumban reported:

*What’s happening in hospitals doesn’t encourage care seekers. As you know, a woman in labour can die just because of a little negligence. But when you get to the hospital, the nurses on duty wait to be bribed before taking care of you. I saw how they treated the wife of my brother and I vowed never to go there. Now, every time I want to give birth, I will go to nah Mariama, a traditional birth attendant in the village.* (R16).

This transformation of maternal healthcare into a commodity, as shown above, has serious consequences for maternal health in the Noun Division. In this area, the maternal health indicators are getting worse, despite an overall improvement in the region. For example, according to statistics from the West Regional Public Health Delegation, the prenatal consultation rate, which was 56% in the early 1990s, had fallen to 48% in 2014. The facility-based deliveries rate also dropped from 68 to 53% during the same period (R14). This under-use of health services by women in labour has had a negative impact on maternal mortality. Moreover, as a healthcare provider in Foumbot noted, “*The Noun Division is the division of the Western Region where most women die during childbirth*” (R17). In this locality, the mortality rate today is 654 deaths per 100,000 live births, against 630 for the whole region. To explain this degradation of maternal health indicators, our respondents were unanimous that the greediness of health providers created inequalities and limited access to maternity services.

## Discussion

The aim of this article has been to show, through empirical methods, the impact of the commodification of healthcare in public maternity units on maternal health in the Noun Division. Thus, during 3 months of immersion in Foumban, Foumbot, Bangourain, and Kouoptamo health districts, we collected data through direct observation and interviews. This triangulation of qualitative methods showed that public maternity units have become, since the 1990s, the scene of ethical abuses, such as diversion of parturients to private healthcare centres, embezzlement of drugs and their resale in parallel systems, medical over-prescription, the racketeering of care seekers, and the subtle request for bribes, among others.

This expansion of anti-values or practices that contravene the principles of medical ethics has fostered serious discrimination in the public maternity services of the Noun Division. In this locality, access to antenatal care and assisted deliveries has become the exclusive privilege of the few women who are able, thanks to their financial and social resources, to overcome the usual indifference of health professionals. Underprivileged women who cannot afford those first class services are kept waiting, against their will, for long hours in the corridors. Even when they are admitted, they are administered perfunctory and inattentive care. Most women, to avoid these frustrations, have elected to use traditional birth attendants in their communities, who assist them from the beginning to the end of their pregnancies.

Although no empirical study has focused specifically on the deviant practices in public maternity services in the Noun Division, this issue has been addressed directly or indirectly in different social contexts. For example, with reference to maternal health services use at the national level, Bonono and Ongolo-Zogo reported that, in Cameroon, even though the utilization level of antenatal care services is 60%, only 35% of pregnant women attend the first trimester visit, notably the one before the end of the first 12 weeks of gestation [9]. This is troubling, as failure to attend this first session has been associated with increased maternal morbidity and mortality. The same authors note that the rate of facility-assisted childbirth is only 61.8% in Cameroon, a country where the government has subscribed to several commitments to improve geographical coverage of care to reduce maternal mortality [9]. To explain this non-optimal use of maternal health services in Cameroon, they point to many factors reported by women interviewed in this research field, such as the indifference of service providers, social inequalities, and the amplification of the bribe phenomenon. With reference to antenatal care, they note: “The anonymous patient is subjected to long waits and additional informal expenses, only to obtain, in the end, a consultation of inferior quality” [9].

The same ethical abuses denounced by our respondents were also criticized in maternity hospitals in West Africa, notably in Benin, Niger, and Senegal, by Olivier de Sardan et al. [10]. These authors described the diversion of drugs that are officially free and their resale to parturients: “To supplement their monthly salaries, nurses move some medicines from official to private pharmacies and sell them to patients who are in need of care” [10]. They also argued that, in their observation context, the hospital was organized in such a way that patients coming to the hospital were propelled (as in the public maternity services in Noun) through a long and complex trajectory where every step entailed unwarranted charges with which patients had to comply to continue their care. Under these conditions, users who are referred by a health professional are spared these hassles, as they are well received and well treated. At the same time, the mass of anonymous and poor patients are left to suffer various forms of contempt, outside the bounds of the rules of decency and modesty that apply in the surrounding society. Their consultations are expeditious, mechanical, inattentive, without dialogue, and without explanation [10], because, as in the Noun Division health sector, a user of health facilities is not considered as a patient, but rather a client.

Without focusing specifically on maternal health, Bayemi wrote about corruption in the hospital sector and its consequences on the management of patients in Cameroon. From interviews conducted with the staff of public hospitals in Douala, this author showed that since the drastic drop in the salaries of civil servants in the mid-1990s, health workers advanced in their daily work, like those observed in Noun Division, through the collection of bribes and the transfer of patients from public to private centres. According to his analysis, these practices, which affect the population health status of the economic capital (Douala) of Cameroon, have led to a drop in public revenues caused by the fact that, as in our research context, public funds are confounded with private ones.

However, while the violation of ethical values in health facilities peaked in Cameroon in the late 1990s, it was already on the scene, widespread and systemic, as stated by Médard. In light of the above, it appears that patients’ financial resources determine their access to maternal healthcare in Cameroon in general and more specifically in the Noun Division. In this country, as Médard showed, healthcare is not at the heart of the relationship between care providers and care seekers. What is most important between these two actors is the exchange of money for healthcare [3]. Thus, as seen in the Noun Division, this trade that has developed in Cameroonian health facilities is the main explanation for the worsening of maternal health indicators, as Bayemi concludes.

Despite the existence of several texts and the multiplication of institutions whose aim is to fight this scourge, it has continued to grow to a large extent in the health sector, as has been shown by Fall and Gueye in the Senegalese context [13]. Under these conditions, pregnant women are compelled to turn to traditional birth attendants in their communities. In addition to limiting the impact of measures taken for the Reduction of Maternal Mortality Campaign in Africa (CARMMA), this discrimination against financially vulnerable women in public maternity services is the main factor behind the worsening of maternal health indicators in this West Region.

Due to medical ethical restrictions and given the methodological approach (direct observation) used in the present study, it was not possible for the researcher to take part in consultations (discussions) between patients and care givers. Consequently, we were limited to reliance on care givers’ and patients’ reports. Moreover, as patients could choose to answer the researcher’s questions or not, many declined to respond, without giving any explicit reasons. This phenomenon had an impact on the number of people actually interviewed, which is by far lower than the number of people contacted.

## Conclusion

In this study aimed at developing knowledge on the impact of the commodification of maternal healthcare on antenatal care and facility-based delivery, it emerged that maternity services have become a real market where providers are almost indifferent to pregnant women who are financially vulnerable. Health professionals in charge of maternal healthcare services deliver attentive care in hospital or at home to privileged women who provide them, through unofficial fees, with additional revenues. Others steal and resell free medicines to parturients and treat these vulnerable users with contempt. In some cases, health professionals even abandon them, whatever their health condition, to look after those who can pay additional fees. These ethical abuses observed in the Noun public health facilities drive women to use, from the beginning of their pregnancies to the delivery, only healthcare delivered by traditional health attendants. This dramatic maternal health situation in this part of the country raises the question: could a pay raise for care providers lead to a reversal of these behaviours in the maternal health sector?

## Abbreviations

CARMMA: Reduction of maternal mortality campaign in Africa
CHWs: Community health workers
CSI: Integrated health centre
IMF: International monetary fund
R: Respondent
SAPs: Structural adjustment programmes
WB: World Bank
WHO: World Health Organization

## Footnotes

1 Deviations here refer to all practices condemned by Article 13 of the Prime Ministerial Decree No. 2004/321 of 08/12/2004 on the Codes of ethics of care givers.

2 This article says: Are forbidden: any commission, all acceptance or sharing of clandestine money between practitioners and patients; the acceptance of commission for any medical act, including for examinations, drug prescriptions, of appliances, sending to a specific clinic, station cure, or health home.

3 These partners are agents who are close to patients, streets sellers, and private pharmacies.

4 New mother in this text designates a woman who has just given birth.
